# Potentially Modifiable Risk Factors For Acute Kidney Injury After Surgery on The Thoracic Aorta: A Propensity Score Matched Case-Control Study

**DOI:** 10.1097/MD.0000000000000273

**Published:** 2015-01-16

**Authors:** Won Ho Kim, Mi Hye Park, Hyo-Jin Kim, Hyun-Young Lim, Haeng Seon Shim, Ju-Tae Sohn, Chung Su Kim, Sangmin M. Lee

**Affiliations:** From the Department of Anesthesiology and Pain Medicine, Samsung Changwon Hospital, Sungkyunkwan University School of Medicine, Changwon, Republic of Korea (WHK, HSS); Department of Anesthesiology and Pain Medicine, Samsung Medical Center, Sungkyunkwan University School of Medicine, Seoul, Republic of Korea (MHP, HJK, H-YL, CSK, SML); and Department of Anesthesiology and Pain Medicine, Gyeongsang National University School of Medicine, Jinju, Republic of Korea (J-TS).

## Abstract

Perioperative risk factors were identified for acute kidney injury (AKI) defined by the RIFLE criteria (RIFLE = risk, injury, failure, loss, end stage) after surgery on the thoracic aorta with cardiopulmonary bypass (CPB) in this case-control study.

A retrospective review was completed for 702 patients who underwent surgery on the thoracic aorta with CPB. A total of 183 patients with AKI were matched 1:1 with patients without AKI by a propensity score. Matched variables included age, gender, body-mass index, preoperative creatinine levels, estimated glomerular filtration rate, a history of hypertension, diabetes mellitus, cerebrovascular accident, smoking history, or chronic obstructive pulmonary disease to exclude the influence of patient demographics, preoperative medical status, and baseline renal function. Multivariate logistic regression analysis was used to evaluate for independent risk factors in the matched sample of 366 patients.

The incidence of AKI was 28.6% and 5.9% of patients from the entire sample required renal replacement therapy. AKI was associated with a prolonged postoperative hospital stay and a higher one-month and one-year mortality both in the entire and matched sample set. Independent risk factors for AKI were a left ventricular ejection fraction <55%, preoperative hemoglobin level <10 g/dL, albumin <4.0 g/dL, diagnosis of dissection, operation time >7 hours, deep hypothermic circulatory arrest (DHCA) time >30 min, pRBC transfusion >1000 mL, and FFP transfusion >500 mL. Although the incidence of poor glucose control (blood glucose >180 mg/dL) was higher in patients with AKI in matched sample, it was not an independent risk factor.

AKI was still associated with a poor clinical outcome in the matched sample. Potentially modifiable risk factors included preoperative anemia and hypoalbuminemia. Efforts to minimize operation time and DHCA time along with transfusion amount may protect patients undergoing aortic surgery against AKI.

## INTRODUCTION

Acute kidney injury (AKI) has been reported to be a common and important complication of cardiothoracic surgery.^1^ The incidence of AKI after thoracic aortic surgery is slightly higher than the incidences after other cardiothoracic surgeries.^1–4^ Moreover, up to 8% of patients require renal replacement therapy (RRT) after thoracic aortic surgery,^2,3,5,6^ and the short-term mortality of these patients is reported to be up to 64%.^3,5,7^

There have been many studies which have identified risk factors for AKI after cardiothoracic surgery.^2–4,7–15^ However, there is a lack of consistency regarding risk factors reported in these studies. Old age, gender, elevated body-mass index (BMI), baseline poor renal function, and a history of hypertension, diabetes mellitus, smoking and cerebrovascular accident were reported to be independent risk factors for AKI.^2–4,7–11^ However, these variables are easily intuited, non-modifiable risk factors, making their clinical significance limited. It is expected that an old patient with multiple comorbidities and poor preoperative renal function may develop AKI postoperatively. Furthermore, these parameters may function as confounders and conceal the clinically useful modifiable risk factors. Therefore, an attempt was made to find modifiable independent risk factors related to thoracic aortic surgery, administration of anesthesia, and preoperative blood test results after excluding potential confounders regarding patient demographics and baseline clinical parameters.

The purpose of this study was to perform a matched case-control study using a propensity score to find independent and modifiable risk factors for postoperative AKI after thoracic aortic surgery controlling for patient demographics, preoperative clinical status, and baseline renal function.

## METHODS

**FIGURE 1:**
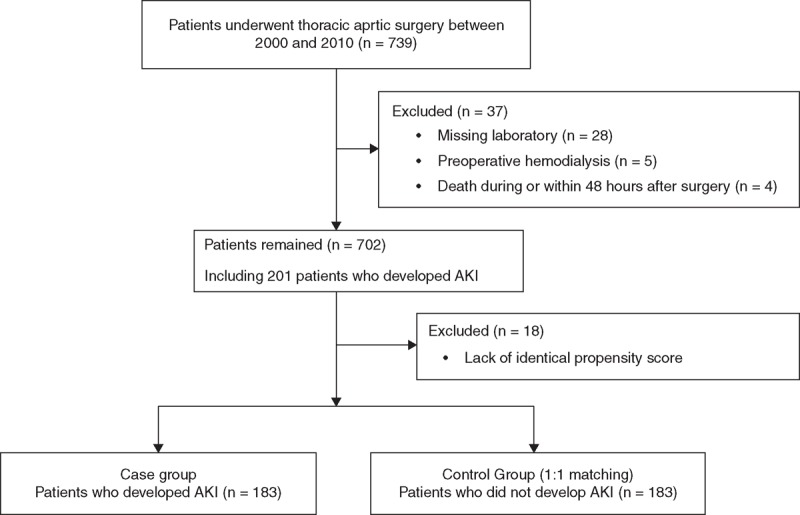
Flow diagram outlining the inclusion and exclusion criteria and study design. AKI = acute kidney injury.

After obtaining Samsung Medical Center Institutional Review Board approval (2011-06-077), the electronic medical records were retrospectively reviewed for 739 patients who underwent surgery on the thoracic aorta with cardiopulmonary bypass (CPB) at the reporting institution between 2000 and 2010. The surgeries included ascending aorta replacement with graft interposition, aortic arch surgery with deep hypothermic circulatory arrest (DHCA), descending aorta replacement for aortic dissection or aneurysm, any surgery with concomitant coronary artery bypass surgery. The need for informed consent was waived for this study given the retrospective design. Patients with RRT before surgery were excluded due to the difficulty of measuring the progression of renal dysfunction (n = 5). Patients were also excluded if they had missing perioperative serum creatinine (sCr) values or urine output values (n = 28), or if they died within 24 hours postoperatively (n = 4). Of the remaining 702 patients, 201 patients developed AKI defined by the RIFLE criteria. A total of 183 patients with AKI were matched 1:1 with those without AKI with the following variables as contributors to the propensity score: age, gender, BMI, and preoperative creatinine levels, estimated glomerular filtration rate (eGFR), a history of hypertension, diabetes mellitus, cerebrovascular accident, smoking history, or chronic obstructive pulmonary disease to exclude the influence of patient demographics, preoperative medical status and baseline renal function (Figure 1). Eighteen patients with AKI were not matched with those without AKI due to the lack of identical propensity scores and these patients were excluded from the matched sample analysis. Overall 183 patients with AKI were compared with 183 patients without AKI.

Demographic or perioperative variables known to be related to postoperative renal dysfunction were included in this study after literature review (Table 1 ). They included preoperative cardiovascular status, surgery-related factors, anesthesia details, and blood test results. sCr concentration has important limitations as a measure of renal function,^16–19^ so eGFR based on the modification of diet in renal disease formula^20^ was also considered as a contributor to the propensity score. Postoperative outcome variables included the need for postoperative RRT, length of hospital stay, and one-month and one-year mortality.

**TABLE 1:**
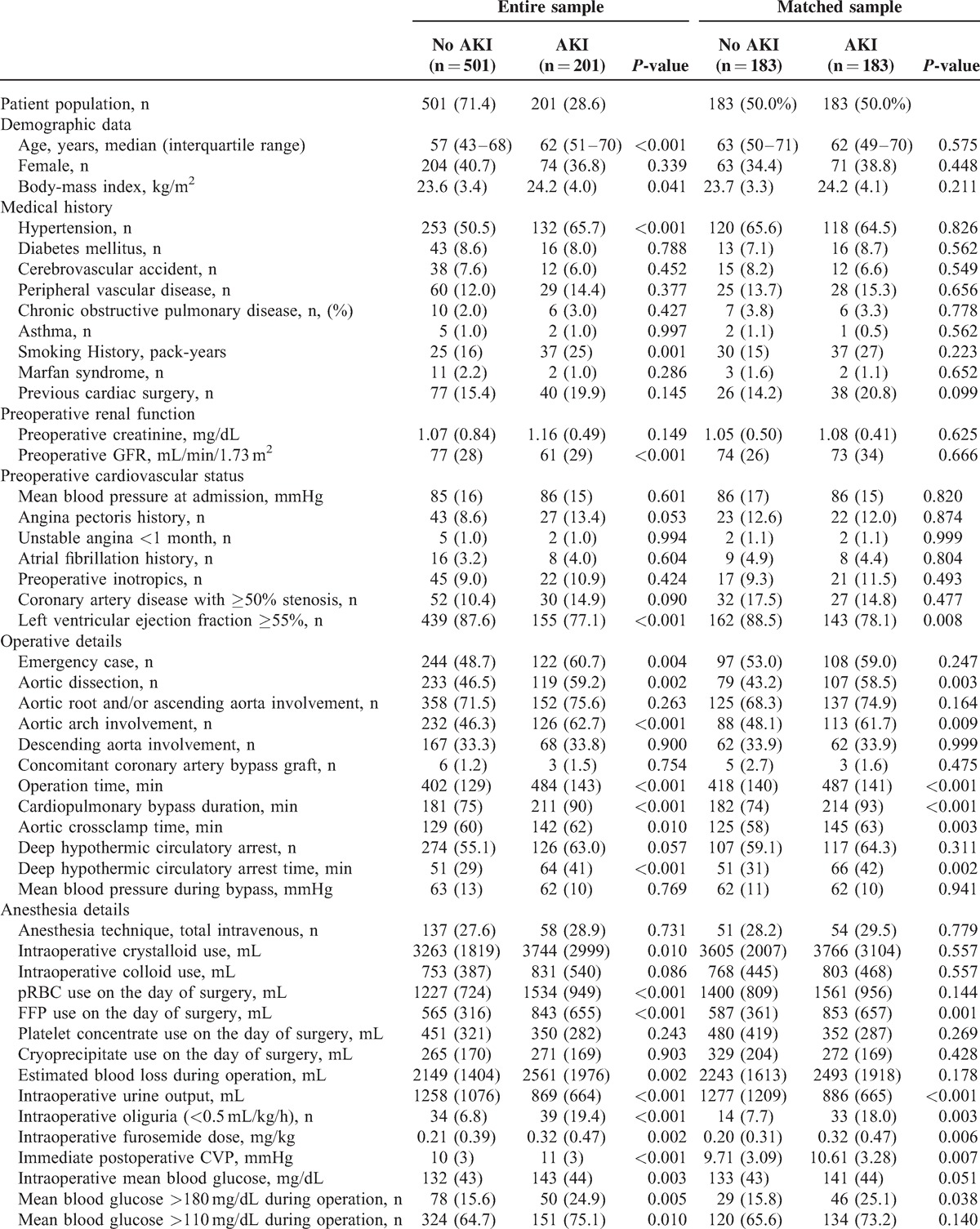
Patients Characteristics and Perioperative Parameters of the AKI and Non-AKI Group in the Entire Sample and in the Matched Sample

**TABLE 1 (Continued):**
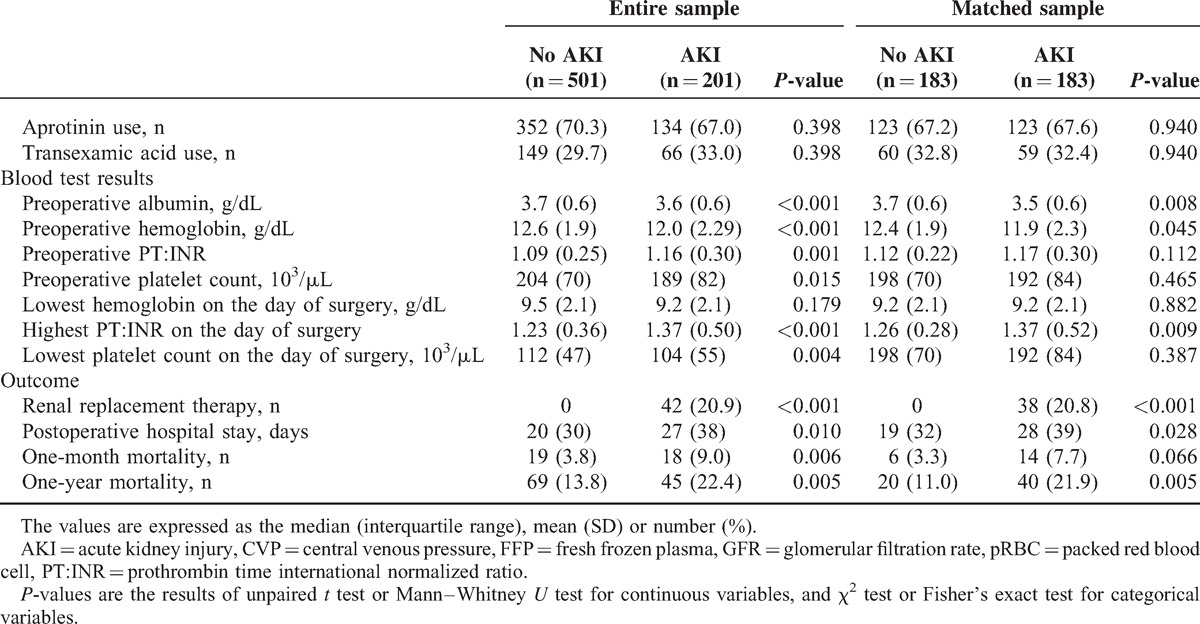
Patients Characteristics and Perioperative Parameters of the AKI and Non-AKI Group in the Entire Sample and in the Matched Sample

The development of postoperative AKI was the primary outcome. AKI was defined according to the RIFLE criteria (RIFLE = *r*isk, *i*njury, *f*ailure, *l*oss, *e*nd stage)^2^ which have been used in patients undergoing cardiothoracic surgeries.^2,6^ The RIFLE criteria classify AKI by severity based on the maximal change in sCr level and eGFR until postoperative day 7 compared with preoperative baseline values. All patients who met the RIFLE criteria for Risk, Injury, and Failure were classified as having AKI. RRT was defined as a new dialysis requirement after surgery. Operative mortality was defined as one-month and one-year mortality.

Anesthesia was maintained either by total intravenous anesthesia or by inhalational agents. Aprotinin or tranexamic acid was used for coagulation support. Since aprotinin was withdrawn from the market due to the BART study,^21^ tranexamic acid has been used to reduce bleeding at the reporting institution. Arterial cannulation was performed in the right axillary, femoral artery, or ascending aorta and venous cannulations were bicaval or right appendage. CPB was routinely instituted at 2.2 to 2.5 L/minutes/m^2^. Selective anterograde perfusion was usually instituted through the right axillary artery with clamping of the innominate and left common carotid arteries to maintain cerebral oximetry saturation within a 10% change of baseline values.

SPSS software version 20.0 (IBM Corp., Armonk, NY) was used to analyze the data. For all analyses, *P* < 0.05 was considered statistically significant. To ensure accurate estimates, the study sample size was determined according to a target number of outcome events of 10 per independent predictor.^22^ For the current study this was estimated to be 400 patients or more, thereby permitting unbiased fitting of 10 or fewer predictor variables in a multiple logistic regression model (estimated 25% incidence of postoperative AKI).^22^ Similarly, 200 or more patients or more were required for a 50% incidence of postoperative AKI for a 1:1 matched sample set.

Categorical variables were reported as an absolute number (n) and relative frequency (%), whereas continuous variables were reported as a mean (standard deviation) or median (interquartile range), as appropriate. The Shapiro–Wilk test was used to determine the normality of the data distribution. Categorical variables were compared between the AKI and no AKI group with the Fisher's exact test or χ^2^ test according to their expected counts. Continuous variables were compared with the unpaired *t* test or Mann–Whitney *U* test according to their normality. Logistic regression models were used to identify univariate and multivariate predictors for AKI in matched samples. Univariate logistic regression analysis was used first to identify possible risk factors for AKI, and the multivariate model included only variables that were significant on univariate analysis (*P* < 0.05). Before performing logistic regression analysis, the cut-off point was determined for the continuous variables on the receiver operating characteristic curve that had the maximal sum of sensitivity and specificity. Variables with commonly used normal values, eg, left ventricular ejection fraction (LVEF), were categorized with their normal cut-off values. The cut-off level for serum albumin was determined according to a previous study.^23^ Predictor variables were selected from a list of 15 candidate variables (Table 2) by performing a backward Wald selection with a significance criterion of *P* < 0.05.

**TABLE 2:**
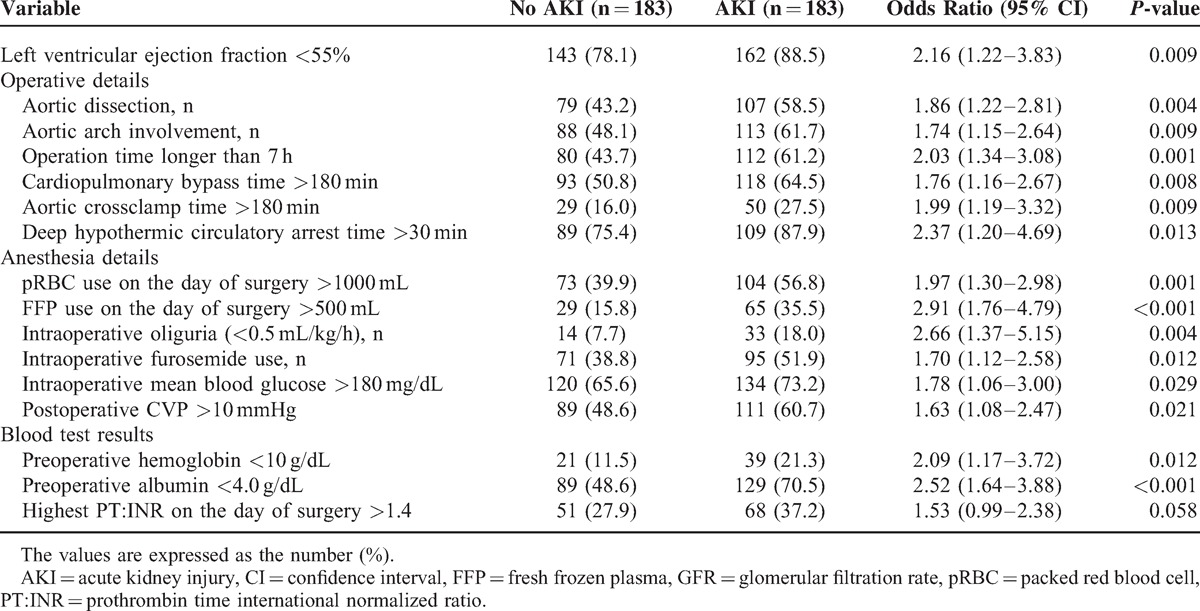
Univariate Analysis of Categorized Risk Factors for Acute Kidney Injury Within all RIFLE Classification in the Matched Sample

Missing data except sCr or eGFR were present in less than 1% of records. Missing values for categorical variables were assigned the most frequent gender-specific value, and continuous variables were assigned the gender-specific median values.

## RESULTS

Among patients who underwent surgery on the thoracic aorta between 2000 and 2010 (n = 739), a total of 702 patients remained after the exclusion of 37 patients. The remaining 702 patients were analyzed (Figure 1).

The incidence of AKI was 28.6% (n = 201/702) and 5.9% (n = 42/702) required RRT during the first 7 postoperative days. The modality used for RRT was continuous RRT in all patients and one-month and one-year mortality among the patients with RRT were 19.5% (n = 8/42) and 34.1% (n = 14/114). Demographics and perioperative variables according to the diagnosis of AKI in both the entire and matched samples are presented in Table 1 . There were differences in demographics, medical history, and preoperative renal function between the patients without AKI and those with AKI in the sample set of all patients. Specifically, the patients with AKI were older and had a higher BMI. They smoked more and had poorer baseline renal function. The matched group set included 183 pairs of patients with and without AKI. As illustrated by the *P* value of unpaired *t* test or Mann–Whitney test, the groups were well-balanced for the variables, which were used for contributors to the propensity score. There were no differences in demographic data, medical history, or preoperative renal function in the matched sample set.

The results of univariate and multivariate analysis of risk factors for AKI within all RIFLE classes in the matched sample set are shown in Tables 2 and 3. Among the 15 potential risk factors determined by univariate analysis, independent risk factors for AKI were LVEF <55%, preoperative hemoglobin level <10 g/dL, albumin <4.0 g/dL, a diagnosis of dissection, operation time >7 hours, DHCA time >30 minutes, pRBC transfusion >1000 mL, and FFP transfusion >500 mL. AKI was associated with a prolonged postoperative hospital stay and a higher one-month and one-year mortality both in the entire and matched sample set (Table 1 ).

**TABLE 3:**
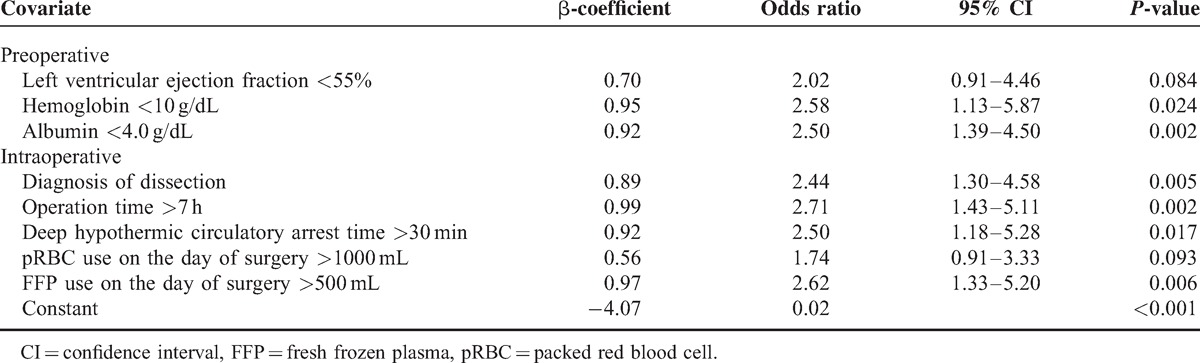
Multivariate Analysis of Risk Factors for the Development of Acute Kidney Injury in the Matched Sample

## DISCUSSION

This propensity score-based matched case-control study was designed to determine independent risk factors for AKI defined by the RIFLE criteria after graft replacement of the thoracic aorta for acute dissection or aneurysm. An attempt was made to find clinically useful, modifiable risk factors for AKI in a matched sample after controlling for demographic and clinical confounders. Modifiable risk factors included preoperative anemia and hypoalbuminemia. Prolonged surgery time along with DHCA time and large transfusion volumes were proven to be independent risk factors. After matching potential or previously proven confounding factors related to patient demographics and baseline medical status, this study could not only identify potentially modifiable risk factors, but also exclude other potentially modifiable risk factors for AKI after aortic surgery including intraoperative hyperglycemia, colloid use and antifibrinolytic agents. There have been few studies which evaluated these risk factors altogether in a matched case-control study.

The incidence of postoperative AKI in the entire sample (28.6%) was slightly lower than that found in previous studies reporting incidences of 43% and 48% using the RIFLE criteria.^1,2^ The one-month mortality in patients with AKI in the entire sample was 9.0%, which was lower than what has been reported in previous studies.^2,5,7^ Forty-two patients (5.9%) required postoperative RRT, which was within a range of 2.1% to 8.0% reported in previous studies.^2–4^

The contributors to the propensity score-based matching were determined based on the previously reported risk factors for AKI after thoracic aortic surgery. Demographic variables previously reported as risk factors for AKI were included such as age,^3,6,7^ gender^7^ and BMI.^3^ A past medical history of hypertension,^2,3,24^ diabetes mellitus,^7,9,10^ smoking history,^9^ and cerebrovascular accident^11^ have also been reported as predictors for AKI and were included here. However, these factors could function as confounders of attempts to find independent risk factors for AKI, and their clinical significance is limited as these are not modifiable. Baseline renal function was also considered as a contributor to the propensity score, because previous studies have reported that preoperative sCr level or eGFR level are risk factors for AKI.^2,7,9,10,12,14^ However, preoperative renal function would likely influence the incidence of postoperative renal dysfunction and baseline renal function is not modifiable, so efforts were made to exclude its influence in the matched sample set.

The result that preoperative anemia is a modifiable independent risk factor is consistent with previous studies.^3,25^ A recent cohort study reported that the risk of AKI increased with the need for transfusion in patients undergoing cardiac surgery, and this risk increased to a greater extent in patients with preoperative anemia than in those without anemia.^26^ The need for transfusion during the surgery was also an independent risk factor in this study, which is consistent with previous studies.^2,9^ Therefore, preoperative correction of anemia and interventions that reduce perioperative transfusion requirements may prevent postoperative AKI.

Preoperative hypoalbuminemia was an independent risk factor in this study, which is consistent with previous studies.^23,27,28^ Preoperative hypoalbuminemia was reported to be a major risk factor for AKI after off-pump coronary artery bypass surgery,^23^ and cardiac transplantation.^27^ Preoperative hypoalbuminemia has been associated with prolonged hospital stay, and increased morbidity and mortality after cardiac surgery.^27,28^ Several studies have reported that serum albumin could have a renoprotective effect by improving renal perfusion, inhibiting apoptosis of renal tubular cells, and promoting the proliferation of renal tubular cells.^29–31^

A long operation time (>7 hours) was identified as another risk factor for AKI in this study, while CPB duration has previously been demonstrated to be a risk factor.^2,3,6–8,14,32^ Although a CPB duration greater than 120 minutes was revealed to be a significant risk factor by univariate analysis, it was not shown to be an independent risk factor in the present study. This may be because DHCA time, which is a thoracic aortic surgery-specific variable, was a stronger independent risk factor for AKI than CPB duration in this sample. The logistic regression model identified DHCA time >30 minutes as an independent risk factor for AKI, which was consistent with a previous study.^24^ Meanwhile, in a previous study reported by Englberger et al,^3^ the use of DHCA was not an independent risk factor. This study did not exclude the influence of patient demographics from the multivariate analysis, and provided increased age, elevated BMI and a history of hypertension as independent risk factors. After adjustment for these potential confounders by propensity score-based matching and multivariate logistic regression analysis in the present study, DHCA >30 minutes remained as an independent risk factor. Hypoxic renal injury during DHCA has been thought to be a risk factor for AKI,^33,34^ so a long DHCA time may result in a higher incidence of AKI. Therefore, strategies to reduce DHCA time and protect the kidney during DHCA are now needed.

There have been controversies over whether aprotinin use is associated with adverse outcomes.^4,21,35^ Since aprotinin was withdrawn from the market due to the BART study,^21^ tranexamic acid has instead been used at the reporting institution. The use of aprotinin or tranexamic acid was not associated with an increased risk of AKI in the present study in both the entire and matched sample sets. A previous study suggested that the administration of aprotinin does not increase the risk of renal dysfunction.^36^

Blood glucose level was examined as a modifiable risk factor. Intraoperative mean blood glucose level was elevated and the incidence of patients with poor blood glucose control (blood glucose >180 or 110 mg/dL) was higher in patients with AKI. The incidence of poor glucose control (blood glucose >180 mg/dL) was still higher in patients with AKI in the matched sample set. Tight blood glucose control (blood glucose <110 mg/dL) was associated with a reduction in the incidence of postoperative hemodialysis and mortality in cardiac surgical patients in previous studies.^37,38^ However, these findings were refuted by a recent multicenter trial and meta-analysis.^39,40^ Since these studies did not consider AKI by the RIFLE criteria, the effect of blood glucose control on AKI should be evaluated in a further study.

This study had several limitations. First, the present study reviewed a relatively small number of patients compared to previous studies.^10,12–14^ However, considering the relatively high incidence of AKI after aortic surgery, the sample size was considered to be sufficient to generate multiple independent risk factors. Eight independent variables were included in our logistic model containing 183 outcome events for 366 patients. The ratio of 22.9 (183/8) events per independent variable is much larger than the suggested ratio of 10.^22^ Second, the data were derived from a single center and thus are limited in external validity. Third, information from the reporting institution was analyzed over 10 years. During this long period the standard of care for aortic surgery has changed, which might alter important covariates used for the prediction of AKI. However, independent variables reported in this study did not change during the study period and a study design using a matching sample set should have overcome this limitation. Fourth, as the present study is a retrospective observational study, this study results cannot demonstrate the causal relationship but only the association of risk factors with AKI. Modifiable risk factors may be the results of chronic illness, not a cause of postoperative AKI. Prospective randomized clinical trials are required to confirm whether the modification of these potentially modifiable risk factors can really reduce the incidence of postoperative AKI.

In conclusion, AKI after surgery on the thoracic aorta was common and was associated with a long hospital stay and high mortality in this matched case-control study. Independent risk factors for AKI included a diagnosis of aortic dissection, decreased preoperative cardiac systolic function, preoperative anemia and hypoalbuminemia, a long surgery time including a long DHCA time, and high transfusion requirements. Correction of preoperative anemia and hypoalbuminemia, efforts to reduce operation time and DHCA time along with reducing transfusion requirements may protect against AKI in patients undergoing thoracic aortic surgery.
